# Radial artery vs saphenous vein graft used as the second conduit for surgical myocardial revascularization: long-term clinical follow-up

**DOI:** 10.1186/s13019-015-0331-9

**Published:** 2015-10-15

**Authors:** Ivana Petrovic, Dusko Nezic, Miodrag Peric, Predrag Milojevic, Olivera Djokic, Dragana Kosevic, Nebojsa Tasic, Bosko Djukanovic, Petar Otasevic

**Affiliations:** Dedinje Cardiovascular Institute and Belgrade University School of Medicine, Belgrade, Serbia

**Keywords:** Radial artery graft, Saphenous vein graft, Clinical outcome

## Abstract

**Background:**

There is ongoing debate regarding the efficacy of the radial artery (RA) as an aortocoronary conduit, with few solid data regarding long-term clinical results. We sought to determine if the use of the RA as the second arterial conduit, beside left internal thoracic artery (LITA), would improve long-term clinical outcome after CABG as compared to saphenous vein graft (SVG).

**Methods:**

Between March 2001 and November 2003, 200 patients underwent isolated CABG and were randomized in 1:1 fashion to receive either LITA and RA grafts or LITA and SVGs. The primary end point was composite of cardiovascular mortality, non-fatal myocardial infarction and need for repeat myocardial revascularization (either surgical or percutaneous).

**Results:**

There was no significant difference in absolute survival, with 12 deaths in each group during the study period (log rank = 0.01, *p* = 0.979). There were 3 and 2 cardiac deaths in RA and SVG groups, respectively. There was no difference in long-term clinical outcome between the groups (log rank = 0.450, *p* = 0.509). Eleven patients in RA group had one or more non-fatal events; 7 patients suffered a myocardial infarction, 9 patients underwent percutaneous coronary angioplasty, and 1 patient required redo coronary surgery. Likewise, 13 patients in SVG group had non-fatal event; 7 patients had myocardial infarction, 13 patients had percutaneous coronary intervention and 3 patients required redo coronary surgery. Angiograms were performed in 23 patients in RA group (patency rate 92 %) and 24 in SVG group (patency rate 86 %) (*p* = 0.67).

**Conclusion:**

In this small randomised study our data indicate that there is no difference in the 8 year clinical outcomes in relatively young patients between those having a RA or a saphenous vein graft used as a second conduit, beside LITA, for surgical myocardial revascularisation.

## Background

Left anterior descending (LAD) coronary artery bypass grafting (CABG) with the left internal thoracic artery (LITA) is regarded as the gold standard in coronary bypass surgery [1]. Initially described in 1973 by Carpantier and colleagues [2] the radial artery (RA) was soon abandoned as a bypass graft as reports documented dismal early angiographic outcomes [2, 3]. However, with various methods to counter spasms, such as a block harvesting, the long-term outcome has improved significantly, and the RA is currently being used as the second graft of choice after the LITA in many institutions [3, 4].

Most literature reports [5–10] demonstrate excellent patency rates in protocol-driven studies and in symptomatic patients. Reported RA patency rates range from 83 to 98 % at 1–7 years after CABG. However, although most clinical and patency reports regarding the RA have been favorable, some are not [11, 12]. Hence ongoing doubt and debate remain regarding the efficacy of the RA as an aortocoronary conduit, with few solid data regarding the medium-term patency rate of these grafts.

The aim of this study was to determine if the use of the RA as the second arterial conduit, beside LITA, would improve long-ter m clinical outcome after CABG as compared to saphenous vein graft (SVG).

## Methods

### Patients

Between March 2001 and November 2003, 200 patients underwent isolated CABG and were randomized in 1:1 fashion to receive either LITA and RA grafts or LITA and SVGs. Use of additional SVGs was permitted in both groups depending on angiographic findings. The study protocol was approved by Institutional Ethics Committee, and investigation conforms to the principles outlined in the Declaration of Helsinki. Written consent was obtained from all patients prior to the procedure.

Patients were included in the study if at least one target vessel for RA/SVG grafting had at least 80 % stenosis, was at least 1.5 mm in diameter, and had no diffuse distal disease. Patients were excluded in the case of a single-vessel disease and if they had undergone any concomitant acquired or congenital cardiac or aortic surgery. Hemodialysis was considered a strong contraindication for RA harvesting due to a concern about the need for possible upper limb dialysis access. The exclusion criteria were a positive Allen’s test, a history of Raynaud’s syndrome or vasculitis. In all cases, before RA harvesting, the adequacy of ulnar compensation was assessed by the Doppler method. The RA was always harvested from the non-dominant arm; bilateral RA harvesting was never performed.

### Procedures

All patients underwent conventional angiography before surgery using retrograde femoral artery catheterization under standard fluoroscopy using an iodine contrast agent. Each angiogram was evaluated by two experienced cardiologists and the decisions were made by consensus. During the follow-up period coronary angiographies were performed if clinically indicated.

Complete echocardiographic examination was performed in all patients prior to index surgery. Left ventricular ejection fraction was assessed using Simpson biplane formula.

All patients were operated at the Dedinje Cardiovascular Institute, Belgrade, Serbia, a tertiary care center. Open harvest of the RA was used in all patients. For the myocardial protection purposes we used 600–1000 ml of cold antegrade modified St. Thomas cardioplegic solution to initially arrest the heart. Topical cooling of the heart was used during procedure (ice slash). Cardioplegia was repeated only if cross-clamp time exceeded 90 min. Cold blood cardiopegia was used only in patients with a EF < 30 %. Side-biting clamps were used for performing proximal anastomoses in all patients. All RA grafts were deployed to the artery with at least 80 % stenosis, providing that it is considered an important coronary artery (smaller, same territory arteries or arteries supplying heavily infarcted areas were not grafted with radial artery). During or after the procedure no intravenous drugs were given to prevent RA spasm. However, we used topically verapamil and nitroglycerin solution (balanced to pH 7.4). All radial arteries were rinsed after harvesting and kept in this solution before implantation. All of our patients were given oral preparations of the calcium channel blockers during one year after surgery to prevent RA spasm.

### Follow-up

Patients were followed for 8 years since index surgery for the composite of cardiovascular mortality, non-fatal myocardial infarction and need for repeat myocardial revascularization (either surgical or percutaneous). Data were collected either by phone or during visits.

### Statistical analysis

The data were entered into an electronic database (Access, Microsoft) and analyzed using the SPSS 16.0 software (SPSS Inc.). Continuous variables were expressed as mean and standard deviations. Categorical variables were expressed as percentages. Dichotomous variables were analyzed using the *χ*^2^ test and Fisher’s exact test, and continuous variables were analyzed using the *t*-test. Binary logistic regression analyses with the fixed entry method were performed in order to identify predictors for RA graft occlusion. The parameters examined were defined, and included established risk factors for coronary artery disease. Accordingly, those parameters with the lowest *p* values in the univariate analysis were entered into the regression model; *p* < 0.05 was considered statistically significant throughout.

## Results

### Baseline data

The baseline demographic and clinical data of the patients are depicted in Table 1. The groups were well balanced with respect to demographic, clinical and angiographic data. Briefly, patients were predominantly males, in their mid-fifties, around 40 % were diabetic, and more than 50 % of patients in both groups had previous myocardial infarction. Mean left ventricular ejection fraction was slightly decreased, and the majority of patients had triple vessel coronary artery disease.

**Table 1:** Baseline characteristics of study patients

	LITA/RA/SVG	LITA/SVG	*P* value
Male/female ratio	73/27	73/27	1.0
Mean age (years)	56.3 ± 6.1	57.1 ± 6.5	0.29
Risk factors and comorbidities			
Diabetes	39 %	43 %	0.56
Smoking	67 %	65 %	0.76
Dyslipidemia	75 %	74 %	0.87
Hypertension	92 %	89 %	0.47
PAD	12 %	14 %	0.67
Previous stroke	3 %	2 %	0.65
COPD	9 %	8 %	0.80
Previous MI	57 %	56 %	0.85
Number of diseased vessels	3.08 ± 0.66	3.14 ± 0.66	0.52
Mean LVEF	48.8 ± 10.7 %	48.0 ± 10.8 %	0.60
Coronary artery disease:			
Left main stenosis	26	24	0.74
Double vessel disease	17	16	0.92
Triple vessel disease	83	83	1.0

Abbreviations: *COPD* chronic obstructive pulmonary disease, *LITA* left internal thoracic artery, *MI* myocardial infarctin, *PAD* peripheral artery disease, *RA* radial artery, *SVG* saphenous vein graft

### Operative and perioperative data

There were no perioperative deaths in both groups. The average number of implanted grafts was similar in patients who received RA or SVG (3.08 ± 0.66 vs 3.14 ± 0.66, respectively). All the patients in both groups had LITA grafting on LAD implanted. Table 2 details placement of RA grafts. Briefly, the majority of RA grafts were placed either on first (50 %) or on second (15 %) obtuse marginal branch. RA graft was never placed to the right coronary artery or diagonal branch if they were previously occluded. Perioperative complications are detailed in Table 3. There were no difference between the groups, with a total of 47 events in RA group and 45 events in SVG group (*p* = 0.89). Although numerous, events were mostly mild and resolved upon instution of adequate therapy. Atrial fibrillation was most frequent adverse event in both groups, followed by pleural effusion and hemorrhage. The average length of index hospitalization was 8 days.

**Table 2:** Distribution of radial artery graft placement

	Radial artery
Diagonal branch	9/100 (9 %)
Ramus intermedius	9/100 (9 %)
First obtuse marginal branch	50/100 (50 %)
Second obtuse marginal branch	15/100 (15 %)
Right coronary artery	17/100 (17 %)

**Table 3:** Adverse events after index surgery

	LITA/RA/SVG	LITA/SVG	P value
TIA/Stroke	3	2	0.65
Sternal dehiscence	1	2	0.56
Radial nerve exploration	1	0	0.31
Leg wound infection	0	2	0.15
Pericardial effusion	4	3	0.70
Pleural effusion	7	6	0.77
Perioperative myocardial infarction	3	4	0.70
Atrial fibrillation	28	26	0.75
Total	47	45	0.77

Abbreviations: *LITA* left internal thoracic artery, *RA* radial artery; SVG, saphenous vein graft

### Follow-up

**Fig. 1:**
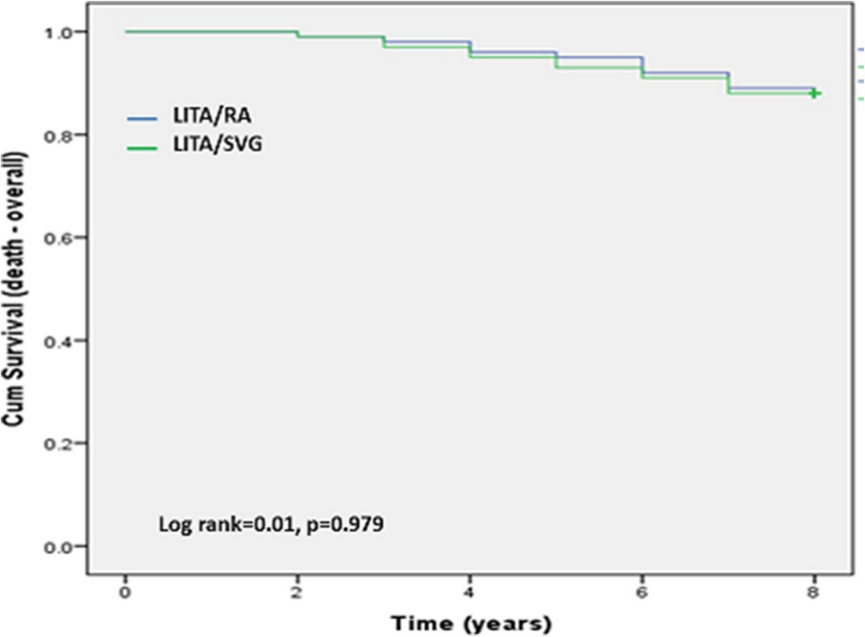
Kaplan-Meier curve for total mortality. Abbreviations: LITA, left internal thoracic artery; RA, radial artery; SVG, saphenous vein graft

**Fig. 2:**
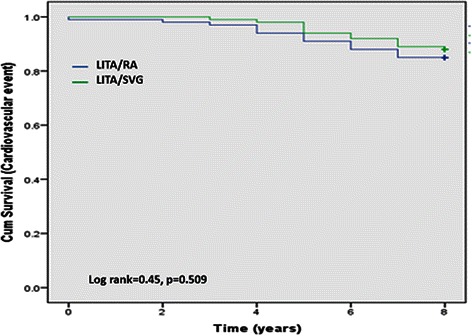
Kaplan-Meier curve for long-term clinical outcomes (composite of cardiac death, non-fatal myocardial infarction and repeat revascularization). Abbreviations: LITA, left internal thoracic artery; RA, radial artery; SVG, saphenous vein graft

All patients were followed for 8 years or until death. There was no significant difference in absolute survival, with 12 deaths in each group during the study period (log rank = 0.01, *p* = 0.979) (Fig. 1). There were 3 and 2 cardiac deaths in RA and SVG groups, respectively. There was no difference in long-term clinical outcome between the groups (log rank = 0.450, *p* = 0.509) (Fig. 2). Eleven patients in RA group had one or more non-fatal events; 7 patients suffered a myocardial infarction, 9 patients underwent percutaneous coronary angioplasty, and 1 patient required redo coronary surgery. Likewise, 13 patients in SVG group had non-fatal event; 7 patients had myocardial infarction, 13 patients had percutaneous coronary intervention and 3 patients required redo coronary surgery.

### Repeated coronary angiography

Repeated coronary angiography was performed in patients who had a positive physical load test or a new coronary event (unstable angina pectoris or myocardial infarction). In RA group 23 underwent repeated coronary angiography, whereas in SVG group 24 patients underwent this procedure. RA graft patency rate was 92 %, whereas SVG patency rate was 86 % (*p* = 0.67).

## Discussion

This study reports on our series of 200 patients undergoing isolated, primary CABG using LITA grafting and the SVG in one group, and RA grafting as the second conduit in the second group. Our data indicate that there is no difference in the long-term clinical outcome between the patients in whom RA or SVG is used as a second conduit, beside LITA, for surgical myocardial revascularization. Additionally, graft patency in patients who underwent coronary angiography was similar between the groups.

### Clinical outcomes

Two randomized clinical trials have reported that event-free survival was greater in patients receiving a radial artery [9, 13]. In the Stand-in-Y trial, event-free survival was similar in patients who received a radial artery compared with a second ITA graft [13]. Two moderately large, single-center observational studies using propensity scores have recently been published [14, 15]. Both early and late survival and event-free survival was enhanced with the use of a radial artery compared with a saphenous vein [15]. Perioperative outcomes including in hospital mortality (0.1 % for the RA patients and 0.2 % for the SVG patients) were similar. Kaplan-Meier survival at 1, 5, and 10 years was 98.3, 93.9, and 83.1 % for the RA group versus 97.2, 88.7, and 74.3 % for the SVG group (log rank, *p* = 0.0011). Cox proportional hazards models showed a lower all-cause mortality in the RA group (hazard ratio 0.72, confidence interval: 0.56 to 0.92, *p* = 0.0084). Ten-year survivals showed a 52 % increased mortality for the SVG patients (25.7 %) versus the RA patients (16.9 %; *p* = 0.0011). For symptomatic patients, RA patency was 80.7 %, which was not different than the LITA patency rate of 86.4 % but was superior to the SVG patency rate of 46.7 % (*p* < 0.001). However, it appears that the use of RA yields inferior long-term clinical outcomes as compared to use of right internal thoracic artery as a second arterial conduit [16].

### Graft patency

There are several reports of the medium to long‐term clinical outcomes for RA grafting. Buxton and colleagues [11] in 2003 reported a prospective randomized study comparing the RA with the free RITA and the SVG. Their 5‐year interim results did not support the hypothesis of superior patency of the RA compared with the RITA or the SVG. The most recent update from the same group in 2010 continued to show no differences in patency rates with pending clinical results [11].

Zacharias et al. [4] in 2004 evaluated the 6‐year clinical outcomes of propensity‐matched patients undergoing LITA‐to‐LAD grafting with either an additional RA graft or SVG as second conduit. In 925 propensity‐matched patients, they found cumulative survival was better with the RA grafts. Angiographic data in restudied symptomatic patients showed a trend toward greater RA graft patency. They reported that the RA graft survival benefit remained when patients were subdivided on the basis of specific risk factors, with women, triple‐vessel disease, younger patients (age ≤65 years), and diabetic patients having a more pronounced survival benefit. However, the 11‐year Kaplan–Meier analysis showed essentially identical RA versus SVG survival for the diabetic patients.

Desai et al. [17] in 2007 the Radial Artery Patency Study, examined randomized angiographic data in 440 RA versus 440 SVG grafts in CABG and showed RA was protective against occlusion, especially in women up to 12 months. A history of peripheral vascular disease was associated with higher risk of RA occlusion, while grafting to a vessel with proximal occlusion improved RA patency. The same group and others in 2008 showed, via angiographic data at 1‐year post‐CABG, that diabetes mellitus was an independent predictor of graft occlusion, although RA grafting was protective in this subgroup versus the SVG [17–21].

The RAPS study is the first multicenter clinical trial reporting radial graft patency beyond 5 years. In the other and larger multicenter CSP 474 VA trial [7], the radial artery or saphenous vein was allocated to the second-best target as determined by the surgeon; they reported that at 1 year, complete graft occlusion was similar in radial and study SVGs (11 %). a 5-year extension is underway. At 5.5 years, the single-center RSVP (Radial Artery Versus Saphenous Vein Patency) study from London, England, reported that complete graft occlusion was markedly less frequent in radial grafts compared with SVGs directed to the circumflex territory [8]. There was no apparent graft-by-territory interaction in the RAPS study, indicating that the relative benefit of the radial artery compared with the saphenous vein applies to both the right and circumflex territories. Graft patency was improved when the radial artery was directed to a more severely narrowed target vessel. The single-center Australian RAPCO (Radial Artery Patency and Clinical Outcomes) study scheduled angiographic follow-up within 5 years in a minority of patients and between 5 and 10 years post-operatively in the majority; they have published interim results from 5 to 10 years of follow-up [16].

Athanasiou et al. [14] included both randomized trials and observational studies in a meta-analysis to compare the patency rates across different follow-up intervals—there were 7 studies with a follow-up >5 years. We updated their review with results from the RAPS study and new data complete to April 2011 [22–25]. Radial grafting was associated with a reduced rate of late graft occlusion compared with saphenous veins (for observational and randomized trials, odds ratio: 0.520, 95 % confidence interval: 0.342 to 0.790, *p* = 0.002; and for randomized trials alone, odds ratio: 0.491, 95 % confidence interval: 0.314 to 0.766, *p* = 0.002.)

When the type of harvest of the RA is concerned, recent prospective, randomized, open-controlled trial that included 119 patients demonstrated that following 5 years of the initial operation both RA harvesting techniques (open and endoscopic harvest ) are safe, effective and result in excellent patency rates [26].

It is very difficult to develop an algorithm for the use of RA as a second conduit for surgical myocardial revascularization. Since it appears that RA is not superior in terms of clinical outcome to the vein grafts for the revascularization of the right coronary artery, we usually use RA for revascularization of the left side system. The main target for RA graft is reasonably sized (≥1.5 mm) obtuse marginal artery with at least 80 % stenosis. However, decision about use of RA graft should be tailored individually in order to achieve greatest clinical benefit for the patient.

### Limitations of the study

The major limitation of the trial is the relatively small number of patients. Additionally, the follow-up duration in our study group was relatively short (8 years), which may lead to the underestimation of net clinical benefit in patients in whom RA graft was used. However, since this was a single-center randomized trial and patients were followed for a considerable time, we believe that a meaningful conclusions may be drawn from our data.

## Conclusion

In this small randomised study our data indicate that there is no difference in the 8 year clinical outcomes in relatively young patients between those having a RA or a saphenous vein graft used as a second conduit, beside LITA, for surgical myocardial revascularisation.
